# Aortitis as a Harbinger of Esophageal Cancer

**DOI:** 10.7759/cureus.20065

**Published:** 2021-11-30

**Authors:** Sandra Ganchinho Lucas, Filipe Alfaiate, Inês Santos, Rita Rocha, Ireneia Lino

**Affiliations:** 1 Internal Medicine, Hospital do Espírito Santo de Évora E.P.E., Évora, PRT; 2 Cardiology, Hospital do Espírito Santo de Évora E.P.E., Évora, PRT; 3 Domiciliary Hospitalization, Hospital do Espírito Santo de Évora E.P.E., Évora, PRT

**Keywords:** large vessel paraneoplastic vasculitis, esophageal carcinoma, aortitis, paraneoplastic syndrome, syndrome of fever of unknown origin, arthritis

## Abstract

Aortitis is a rare diagnosis that requires a high index of suspicion due to nonspecific symptoms and its multiplicity of etiologies.

An 80-year-old man, independent in activities of daily living (ADLs), had three consecutive hospitalizations in three months for fever, general malaise, anorexia associated with arthritis of the hands and feet with the inability to walk. Inflammatory markers were increased without a focus of infection identified. Upper digestive endoscopy (UDE), colonoscopy, blood cultures, thoracoabdominal-pelvic CT and transthoracic (TT) echocardiogram were performed without changes, with discharge for consultation after demonstrating apyrexia. At the patient second hospitalization for fever and arthritis, a transesophageal echocardiogram was performed that showed the presence of multiple complex atherosclerotic plaques, with associated thrombi in all segments of the aorta with a suspicious mass of vegetation on the aorta. Thoracic-abdominopelvic CT demonstrated calcified atheromatosis of the entire aorta with para-aortic nodes; MRI showed aortic thickening; and autoimmunity study was negative. Aortitis was the working diagnosis of possible infectious etiology and anticoagulation and antibiotic therapy were started. Fever recurred and a third admission led to a working diagnosis of inflammatory, non-infectious aortitis. The patient responded well to empiric corticosteroids.

The patient followed up in consultation, remained asymptomatic under a low dose of corticosteroids with negative temporal artery biopsy. In the sixth month, he repeated UDE due to dysphagia, which showed the presence of esophageal neoformation with the histological diagnosis of squamous cell carcinoma, maintaining on the CT as alterations in the aorta.

This is an unusual case of aortitis associated with arthritis with improvement after corticosteroids, which interestingly occurred before the progression of esophageal cancer. In hindsight, we think this may have been a large vessel paraneoplastic vasculitis that preceded the detection of esophageal squamous cell carcinoma.

## Introduction

Aortitis is a rare diagnosis that requires a high level of suspicion [1] due to its presentation of nonspecific symptoms, including pain, fever, headache fatigue, tiredness, malaise, and arthralgias, and its multiplicity of aetiologies [2-4]. The etiology of aortitis can broadly be classified as infectious or non-infectious conditions, or idiopathic [1, 5]. Although all three classifications describe the pathological inflammation of the aortic wall, there are differences in the pathogenesis and treatment [1, 4, 5]. The most frequent infectious causes of aortitis are *Staphylococcus*, *Enterococcus*, *Streptococcus*, and *Salmonella *species, *Mycobacterium tuberculosis*, and *Treponema pallidum*, while the non-infectious causes are large-vessel vasculitis, including giant cell arteritis, and Takayasu arteritis [1, 3, 4]. Therapeutic approaches used for non-infectious aortitis are various as antibiotics have little or no benefit in the treatment of non-infectious aortitis [5]. Furthermore, rarer differential diagnoses, such as aortitis secondary to neoplasms [6] must be taken into account as they require specific care that sometimes differs from the treatment of inflammatory aortitis [2].

This clinical case reports aortitis associated with arthritis that preceded the progression of early esophageal carcinoma.

## Case presentation

Our patient was an 80-year-old male, leukodermic, with a history of adenocarcinoma of the prostate, radiation proctitis, hyperuricemia, and iron deficiency anemia. He came to the emergency department (ED) due to neurological changes with speaking impediments, prostration, hypotension, myalgias, chills, and fever of 39ºC. He had been medicated in the previous two days by an assistant physician with amoxicillin/clavulanic acid without clinical benefit. On admission, a systolic murmur III/VI audible in the aortic area, hypotension, a 39ºC fever, and malaise associated with episodes of prostration.

The first laboratory investigation in ED revealed the following: hemoglobin 13.1g/dL, leukocytes 9700 µL, neutrophils 82% (8000 µL), platelets 221,000 µL, international normalized rate (INR) 1.16, urea 72 mg/dL, creatinine 1.58 mg/dL, and C-reactive protein (CRP) 28 mg/dL. Still in the ED, a lumbar puncture was performed, and the patient started antibiotic therapy, followed by patient admittance to the internal medicine department with a diagnosis of fever of unknown origin and possible central nervous system (CNS) infection for which ceftriaxone and ampicillin were administered for seven days.

**Figure 1:**
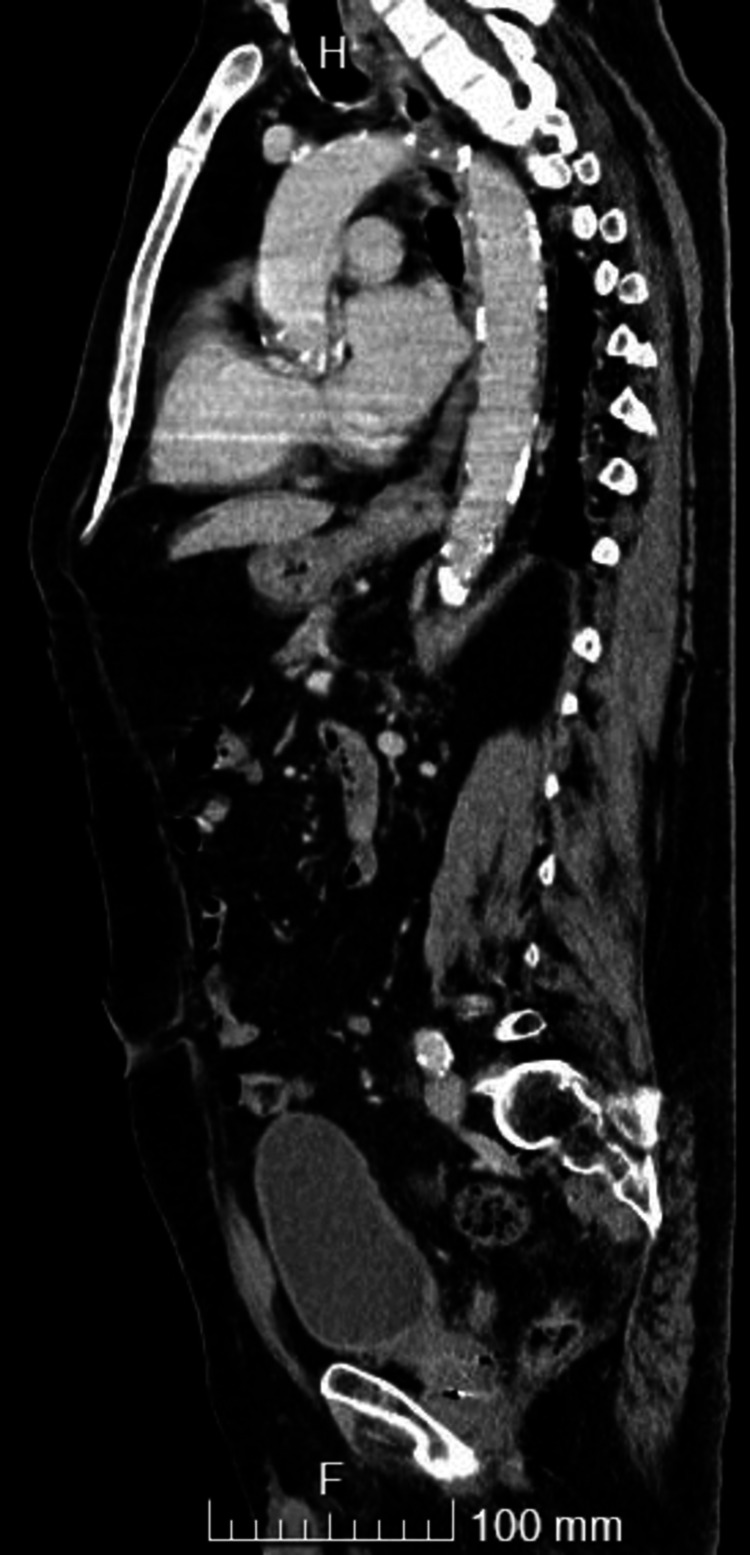
Thoracoabdominal-pelvic CT: calcified atheromatosis of the entire aorta.

During his hospital stay, the patient presented CRP with consecutively lower values, electrophoresis of proteins (SPEP) without monoclonal spike and serum immunoelectrophoresis (SIEP) without changes and serial assay of negative procalcitonin, four blood cultures in antibiotic window and a urine culture, a CSF analysis: protein 61 mg/dL, cell count 0.8 cells/mm3 and negative for bacteriological and neurotropic virus research, negative microbiological and parasitological study of stools as well as negative serology for *Brucella*, *Borrelia burgdoferi*, *Coxiella burnetti*, *Rickettsiosis *(*conori*, *typhi*, *Ehrlichia chaffeensis*), *Treponema pallidum*, Rapid Plasma Reagin (RPR), HIV, and IgG and positive serology for *Campylobacter jejuni *and *Listeria monocytogenes*. Prostate-specific antigen (PSA) was negative, upper digestive endoscopy (UDE), colonoscopy, cranial CT, chest X-ray without alterations, and transthoracic echocardiography (TTE), which showed mild aortic stenosis and an eccentric mitral regurgitant jet without other alterations. The patient continued to have peaks of fever in the afternoon after seven days of antibiotic therapy, so the therapy was suspended due to a lack of clinical and laboratory evidence of infection. After apyrexia was maintained, the patient was discharged to continue follow-up evaluation on an outpatient basis. The patient then underwent a thoracoabdominal-pelvic CT (Figure 1), which identified calcified atheromatosis of the entire aorta with para-aortic nodes.

**Figure 2:**
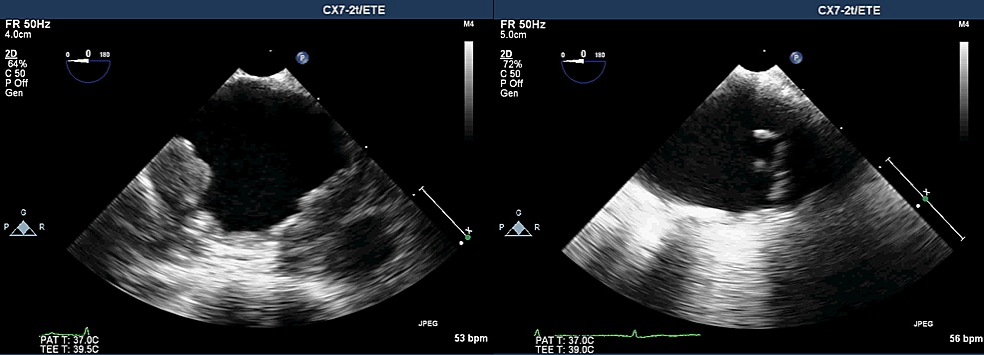
TEE multiple complex atherosclerotic plaques with associated thrombi in the descendent aorta (left) and vegetating mass of erratic movements (7x1.5 mm) in the transition of aortic arch (right). TEE: transesophageal echocardiography

The patient was readmitted after one month due to the same symptoms as previously associated with arthritis of the hands (metacarpophalangeal [MCP] and wrists) and knee without synovitis palpable. During hospitalization, the patient manifested migratory arthralgia. A physical examination, showed no oral ulcers, cutaneous and urogenital lesions, but revealed a systolic murmur, afternoon febrile peaks with a maximum temperature of 38.7°C, normotensive with no differences in measurement between the limbs; a neurological examination and ophthalmological evaluation were normal. A transesophageal echocardiography (TEE) (Figure 2) was conducted, revealing the presence of multiple complex atherosclerotic plaques with associated thrombi in all segments of the aorta with a vegetating mass of erratic movements that did not allow the exclusion of aortic vegetation (7x1.5mm) in the transition of the aortic arch and descendent aorta, while an MRI showed aortic thickening and calcified atheromatosis of the thoracic, abdominal aorta and aortic branches, including visceral branches and supra-aortic trunks. Aortitis was identified based on CT, TEE, and nuclear magnetic resonance imaging (NMR) results, for which antibiotic therapy with vancomycin and gentamicin was started for 12 days. The patient underwent a new TEE at the end of the antibiotic therapy cycle, which gave the same results as the initial one, and was started on anticoagulation with rivaroxaban. During the patient’s hospitalization, the following procedures were performed: analysis of urine culture, four blood cultures with prolonged incubation negatives and anti-nuclear antibodies (ANA), double-stranded DNA (dsDNA), complement C3, C4, IgG, IgG4, and rheumatoid factor without alteration, erythrocyte sedimentation rate (ESR) 37 mm/h, and interferon-gamma release assays (IGRA) tests, serology for hepatitis B and C negative, serial dosing of procalcitonin negative. His maximum CRP was 21.8 mg/dL. He started therapy with naproxen with an improvement of his condition and was discharged with a tight follow-up.

The patient was readmitted 15 days after discharge due to evening fever, anorexia, and inability to walk due to arthritis of the hands, knees, and feet, but without associated malaise or prostration. A hypothesis of large vessel disease such as giant cell arteritis or Takayasu arteritis was considered, and empiric corticosteroid therapy was initiated with prednisolone at 60 mg dosage. Dramatic improvement of the patient’s condition was noted within two days, when he started walking without pain, the patient was discharged on the fifth day to continue follow-up with prednisolone at 20 mg dosage.

Due to the patient's arthritis, he could not tolerate tapering below prednisolone dose of 15 mg after the initiation of corticosteroids, and was maintained until his death. A temporal artery biopsy that was requested, but only performed two months after the initiation of corticosteroids, came out negative.

**Figure 3:**
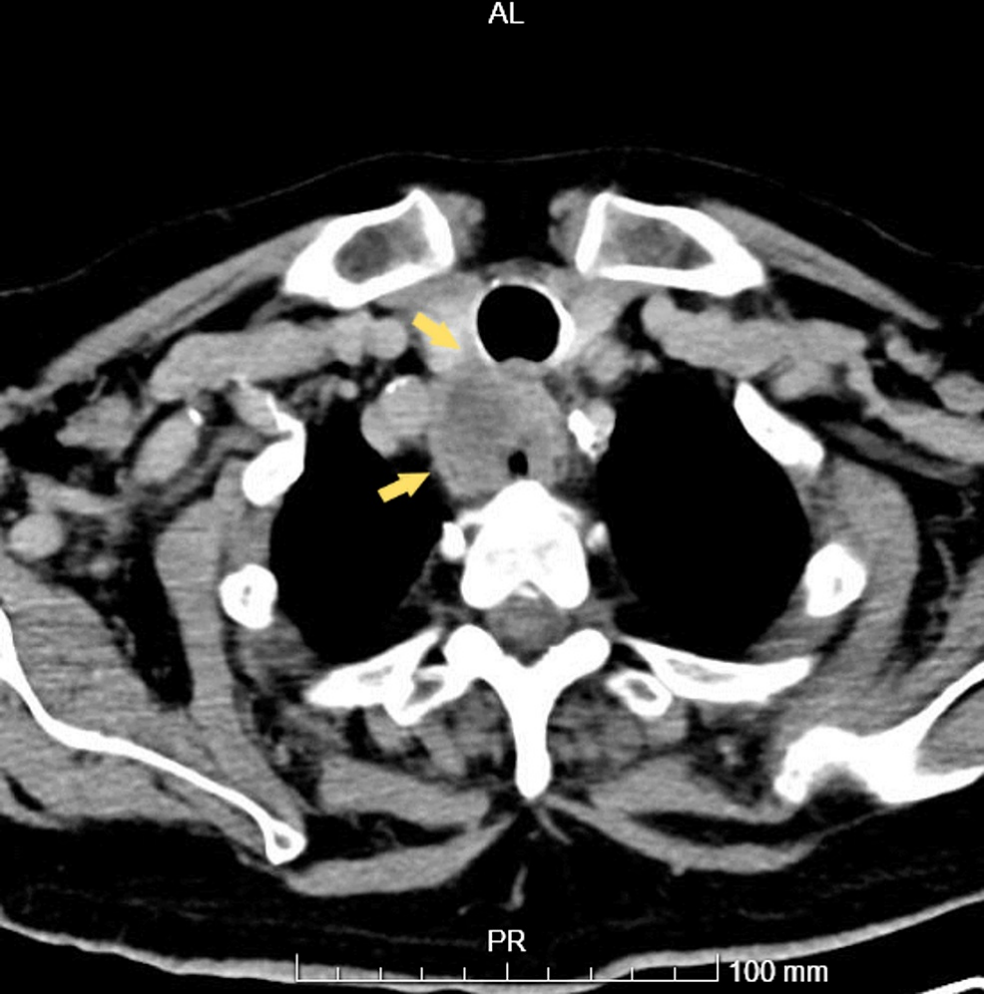
Thoracic CT: Esophageal mass merged with the trachea.

After six months, the patient started to experience dysphagia, therefore, we repeated the UDE, which showed at 17 cm from the upper dental arch the presence of an exophytic and ulcerated esophageal neoformation with a histological diagnosis of necrotic and ulcerated cellular squamous cell carcinoma. A thoracic-abdominopelvic CT (Figure 3) showed a mass without a cleavage plane within the trachea, maintaining the alterations previously described in the aorta. With the advantage of hindsight, a reassessment of the initial CT, the imagiologist described a possible esophageal lesion of small dimensions. The patient started chemotherapy and radiotherapy, as the location of the carcinoma had no indication for surgery, and he died a few months later. A post-mortem examination was refused by the family.

## Discussion

Diagnosis of aortitis remains a challenge, especially when symptoms are atypical, and it is difficult to establish a diagnosis through a biopsy, which is the gold standard, although it is rarely performed [5, 7, 8]. CT, ultrasound, and NMR imaging were decisive in the diagnosis of aortitis as described in the literature [1, 3, 7]. Initially, was considered infectious aortitis due to the described mortality of 21% to 44% [2, 9] fastidious bacteria were considered and blood cultures with prolonged incubation in antibiotic window were performed simultaneously with the neoplasia. Subsequently, the patient was screened for the most frequent inflammatory diseases, given the association between aortitis and large vessel vasculitis. Behçet disease was considered although there were no oral ulcers, cutaneous and urogenital lesions and gastrointestinal ulcers documented by the UDE and colonoscopy without alterations. G4-related disease was not completely excluded since the aortic biopsy was not performed due to the high risk associated, and normal IgG4 values were registered. The temporal artery biopsy results may have masked giant cell arteritis since it was performed under corticosteroids treatment. The Takayasu arteritis [10, 11] and giant cell arteritis [8] diagnostic criteria, according to the American College of Rheumatology and Ishikawa, and despite the clinical improvement of the patient’s arthritis and his general condition under corticosteroids, the patient remained a diagnostic challenge in agreement with inflammatory causes.

Paraneoplastic syndromes can be synchronous, but they can also precede or follow the diagnosis of neoplasia and are defined as clinical alterations that cannot be attributed to direct tumor invasion or metastases [12]. Currently, paraneoplastic syndromes are attributed to the secretion of proteins or hormones or to immunological cross-reactivity [12]. An increased risk of rheumatic diseases triggered both by chronic inflammation caused by the neoplasm and autoimmunity resulting from the anti-tumor immune response has been documented [13].

In the present case, the temporal relationship between esophageal cancer and unfocused fever, aortitis, and later migratory arthritis suggests a paraneoplastic origin of systemic vasculitis. In fact, aortitis may be an early manifestation of systemic vasculitis [14]. Therefore, esophageal carcinoma may be related to aortitis and aortitis-related symptoms, therefore, research was needed to strengthen this hypothesis. A literature review was made by searching though the PubMed® and Google Scholar databases for case reports (English language articles only) using the following search terms: “neoplastic aortitis", “aortitis”, “large vessels vasculitis” and “aortitis and “malignancy”. The reference lists of the selected articles were also considered in the search. To identify if the cancers have ever been attributed to inflammatory aortitis paraneoplastic, from the search results we excluded cases of infectious aortitis, aortitis secondary to the treatment of neoplasms and aortitis secondary to hematologic neoplasms already well described [15]. In the literature, we found only two cases of cancer associated with inflammatory aortitis paraneoplastic colon adenocarcinoma [14], lung carcinoma [16]. No reference was found for inflammatory aortitis paraneoplastic and esophageal squamous cell carcinoma in the literature.

To identify the paraneoplastic vasculitis that has been attributed to esophageal squamous cell carcinoma, a second search was performed for “vasculitis”, “Paraneoplastic syndromes” and “esophageal”, using the same search criteria as the first search. After the search, we verified the results excluding all that didn’t match with esophageal squamous cell carcinoma.

We found only two cases of an association between esophageal cancer and vasculitis paraneoplastic without mentions of large vessel vasculitis or aortitis, one report of an esophageal squamous cell carcinoma [15] and one case of vasculitis [17] in a patient with esophageal cancer that was listed in the table of a case report and review, but the report lacked more details.

## Conclusions

We present an unusual case initially concerning of fever of unknown origin and aortitis, and also arthritis with improvement after corticosteroids treatment that manifested over the development of the clinical case. Curiously the disease developed during the progression of esophageal cancer. Esophageal cancer was not present in the in-depth study carried out initially, but in hindsight, we think this may have been a large vessel paraneoplastic vasculitis that preceded the detection of an esophageal squamous cell carcinoma. Certainly, paraneoplastic large vessel paraneoplastic vasculitis is rare, but associated with the patient’s atypical symptoms, the possibility of a concomitant malignant neoplasm should be considered, which can have a dramatic impact on both morbidity and mortality. As far as we know, this is the first report of panaortitis as a possible association with early-stage esophageal cancer.
